# Television Advertising and Health Insurance Marketplace Consumer Engagement in Kentucky: A Natural Experiment

**DOI:** 10.2196/10872

**Published:** 2018-10-25

**Authors:** Paul R Shafer, Erika Franklin Fowler, Laura Baum, Sarah E Gollust

**Affiliations:** 1 Department of Health Policy and Management Gillings School of Global Public Health University of North Carolina at Chapel Hill Chapel Hill, NC United States; 2 Government Department Wesleyan University Middletown, CT United States; 3 Wesleyan Media Project Wesleyan University Middletown, CT United States; 4 Division of Health Policy and Management School of Public Health University of Minnesota Minneapolis, MN United States

**Keywords:** advertising, Affordable Care Act, enrollment

## Abstract

**Background:**

Reductions in health insurance enrollment outreach could have negative effects on the individual health insurance market. Specifically, consumers may not be informed about the availability of coverage, and if some healthier consumers fail to enroll, there could be a worse risk pool for insurers. Kentucky created its own Marketplace, known as kynect, and adopted Medicaid expansion under the Affordable Care Act, which yielded the largest decline in adult uninsured rate in the United States from 2013 to 2016. The state sponsored an award-winning media campaign, yet after the election of a new governor in 2015, it declined to renew the television advertising contract for kynect and canceled all pending television ads with over a month remaining in the 2016 open enrollment period.

**Objective:**

The objective of this study is to examine the stark variation in television advertising across multiple open enrollment periods in Kentucky and use this variation to estimate the dose-response effect of state-sponsored television advertising on consumer engagement with the Marketplace. In addition, we assess to what extent private insurers can potentially help fill the void when governments reduce or eliminate television advertising.

**Methods:**

We obtained television advertising (Kantar Media/Campaign Media Analysis Group) and Marketplace data (Kentucky Health Benefit Exchange) for the period of October 1, 2013, through January 31, 2016, for Kentucky. Advertising data at the spot level were collapsed to state-week counts by sponsor type. Similarly, a state-week series of Marketplace engagement and enrollment measures were derived from state reports to Centers for Medicare and Medicaid Services. We used linear regression models to estimate associations between health insurance television advertising volume and measures of information-seeking (calls to call center; page views, visits, and unique visitors to the website) and enrollment (Web-based and total applications, Marketplace enrollment).

**Results:**

We found significant dose-response effects of weekly state-sponsored television advertising volume during open enrollment on information-seeking behavior (marginal effects of an additional ad airing per week for website page views: 7973, visits: 390, and unique visitors: 388) and enrollment activity (applications, Web-based: 61 and total: 56).

**Conclusions:**

State-sponsored television advertising was associated with nearly 40% of unique visitors and Web-based applications. Insurance company television advertising was not a significant driver of engagement, an important consideration if cuts to government-sponsored advertising persist.

## Introduction

In late August 2017, the Trump administration announced plans to cut federal funding for Marketplace advertising by 90% for the 2018 open enrollment period, including the elimination of all television advertising [1]. This was the latest upheaval for the Affordable Care Act (ACA) in a tumultuous first year of the Trump presidency, which included an executive order to “minimiz[e] the economic burden” of the law, unplanned advertising reductions at the end of the 2017 open enrollment period, failed legislative efforts to repeal and replace, shortening of the open enrollment period, cuts to enrollment assistance, discontinuation of cost-sharing reduction payments, and a repeal of the individual mandate included in the tax bill signed into law in December [2-6]. The Marketplace, as the combination of state-based and federally run health insurance exchanges created by the ACA is known, provides consumers with the opportunity to compare plans, determine eligibility for financial assistance and Medicaid, and enroll in coverage. The ACA relied on the new Marketplace, the expansion of the Medicaid program (among states that chose to do so) to low-income childless adults, and extending insurance coverage eligibility for youth up to age 26 on their parents’ plans as the main mechanisms to expand access to health insurance in the United States to approximately 20 million people [7].

Thus far, limited evidence on the impact of Marketplace advertising on enrollment and related outcomes suggests that advertising works, though this evidence is not causal. Higher exposure to advertising has been associated with improved perceptions of and knowledge about the ACA. Among a sample of low-income adults in Arkansas, Kentucky, and Texas, those reporting greater exposure to positive advertising about the law were significantly more likely to say that the “ACA helped me” [8]. In a nationally representative sample, those with higher volumes of insurance-related advertising in their media market were significantly more likely to report feeling informed about the ACA and have more positive views of the law [9]. The two published studies to date that have examined the relationship between the dose of Marketplace advertising and enrollment-related outcomes have found that counties with more advertising saw larger decreases in their uninsured rate and a greater likelihood of shopping for and enrolling in a Marketplace plan during the 2014 open enrollment period [10,11]. California has credited aggressive marketing with higher take-up of coverage and lower risk scores, providing a substantial return on investment in terms of lower premiums [12]. As of yet, however, no published research has examined how changes in advertising volume from multiple sponsors over time and within open enrollment periods correspond with consumer engagement with the Marketplace.

Kentucky is an important case for examining these marketing and consumer engagement dynamics. Kentucky created its own Marketplace, known as kynect, and adopted Medicaid expansion under the ACA, which yielded the largest decline in adult uninsured rate in the country from 2013 to 2016 [13]. The state sponsored an award-winning media campaign and also received national recognition for its implementation of the information technology infrastructure for kynect [14,15]. Yet, after the election of a new governor in 2015, state politics led to changes in kynect messaging and promotion that foreshadowed what was to come at the federal level. Specifically, the Bevin administration declined to renew the advertising contract for kynect and canceled all pending television ads with over a month remaining in the 2016 open enrollment period [16]. The objective of this study is to examine the stark variation in advertising across multiple open enrollment periods in Kentucky and use this variation to estimate the dose-response effect of state-sponsored advertising on consumer engagement with the Marketplace. In addition, we assess to what extent private insurers can potentially help fill the void when governments reduce or eliminate television advertising.

## Methods

### Data

We used data from Kantar Media/Campaign Media Analysis Group (CMAG) to measure the volume of local broadcast and national cable television advertising for health insurance and from the Office of the Kentucky Health Benefit Exchange (KHBE) to describe consumer engagement with kynect. The Kantar Media/CMAG data—obtained through collaboration with the Wesleyan Media Project—track airings of individual televised advertisements, including date, time, sponsor, station, and media market, for the period of October 1, 2013, through January 31, 2016, spanning the 2014 to 2016 open enrollment periods. We obtained these data for all 10 media markets in Kentucky, including border markets that reach across state lines (only 2 markets are fully contained within the state). We categorized advertisements into one of 6 sponsor types: (1) kynect; (2) healthcare.gov; (3) insurance companies (eg, Aetna and Cigna); (4) insurance agencies (eg, Healthmarkets Insurance Agency); (5) nonprofits; and (6) other state governments (to capture ads from neighboring states aired in Kentucky). Multimedia Appendix 1 provides a list of ad sponsors and their assigned sponsor type. We identified healthcare.gov advertising through health insurance ads paid for by the US Department of Health and Human Services. A small percentage (565/10,089, 5.6%) of these ad airings were not specific to healthcare.gov, but nearly all either mentioned the “health care law” or explicitly tagged healthcare.gov. Only 0.5% (46/10,089) of these airings had no mention of the ACA at all (ads for Medicare open enrollment). We collapsed the data by sponsor type and week (Sunday to Saturday) in each media market and then calculated a population-weighted average across media markets using media market population estimates from Polidata to provide a state-level estimate of ads aired per week for each sponsor type [17].

The KHBE analytic data were derived from a set of reports from the state to the Center for Consumer Information and Insurance Oversight at Centers for Medicare and Medicaid Services that were obtained through state public records request. Of the 122 weeks between October 1, 2013, and January 31, 2016, there was only a single week (February 9-15, 2014) for which a report was not available and 2 other reporting periods (December 13-19, 2015, and June 30-July 28, 2015) for which one of the outcomes that we used in our analysis (calls) was not reported. We used linear interpolation to impute those missing values. The time periods for each report varied and were often longer outside of open enrollment (eg, every 4 weeks compared with every week within the open enrollment period). To maintain consistent measures, we converted data from the longer reporting periods back to a weekly frequency by averaging the incremental activity over the appropriate number of weeks. For example, if there were an incremental gain of 1000 unique visitors over a 4-week reporting period, we converted this back to 4 individual weeks with 250 unique visitors each week. There were a few reporting periods that had a length other than 7 days (eg, 5, 6, and 8) that we could not standardize (eg, the first week in the data, October 1-5, 2013). We addressed this by including the number of days in the reporting period as a covariate in all regression models.

### Outcomes

The process of shopping for and enrolling in coverage generally involves visiting the Marketplace website to view plans and pricing with the ability to learn about subsidy eligibility (based on family structure and household income) and then select a plan in which to enroll. For this reason, we focused on both parts of the process. First, we assessed how well television advertising does at driving consumers to visit the kynect website. We could not explicitly examine conversion of those website visits into enrollment, but we assessed how television advertising is associated with application submissions and plan enrollment. We used 7 measures as outcomes to represent 2 domains of consumer engagement with kynect—information-seeking behavior and enrollment activity. Our measures of information-seeking behavior were (1) calls to the kynect call center; (2) page views (number of individual pages viewed); (3) visits (including repeats from the same internet protocol address); and (4) unique visitors (excluding repeats) for the kynect website. Our measures of enrollment activity were (5) number of applications completed through Web; (6) total number of applications completed; and (7) number of individuals enrolled in qualified health plans (net of plan terminations). All outcomes were defined as incremental state-week totals (as described in Data above). The advertising data (reported at the weekly level) were then merged with these outcome data at matching time periods.

### Statistical Analysis

We used linear regression models to identify variation in each outcome that was associated with variation in the television advertising volume, with the unit of analysis as a state-week. This approach has been used to analyze consumer engagement with health education campaigns, including the *Tips From Former Smokers* campaign by the Centers for Disease Control and Prevention [18,19]. Each model included the number of ads aired for each of the 6 sponsor types, an indicator for open enrollment periods, and their interaction to allow us to identify the differential effect of advertising during open enrollment. We used a single indicator for all open enrollment periods rather than period-specific indicators (ie, indicators for the 2014, 2015, and 2016 open enrollment periods separately) owing to our limited sample size (122 state-weeks). As such, there may be differential effects related to specific open enrollment periods that we are averaging over. In addition, we included indicators for the weeks of Thanksgiving and Christmas and indicators for the 2 weeks preceding open enrollment (to capture any early plan shopping behavior), the first 2 weeks of open enrollment (to capture early enrollees), and the last 2 weeks of open enrollment (to capture later enrollees). Moreover, we controlled for the number of days in the reporting period as noted above. Average marginal effects were calculated to describe the incremental effect of an additional advertisement per week for each sponsor type. Next, we estimated the share of consumer engagement that was attributable to advertising. To do so, we used linear predictions from each model with the observed advertising during the first 2 open enrollment periods compared with the counterfactual—the absence of kynect or insurance company advertising. This involves using the coefficient estimates from the regression models fitted to the observed data to predict what the outcomes would have been if we set either kynect or insurance company advertising to zero during open enrollment and then comparing those results to the predictions from the model using the actual observed data. All models were estimated with robust standard errors, and *P*<.05 was used as the threshold for statistical significance. Multimedia Appendix 2 shows a correlation matrix for our independent variables. All analyses were conducted in Stata 14.2 [20].

## Results

**Figure 1:**
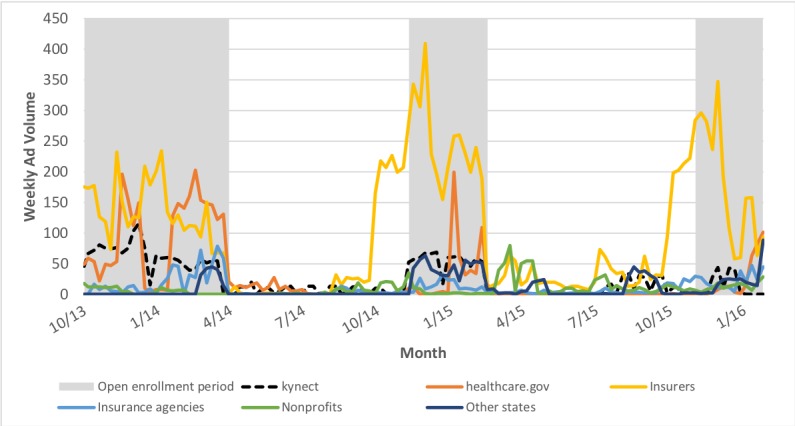
Weekly advertising volume by sponsor type, Kentucky, October 1, 2013-January 31, 2016.

Weekly advertising volume varied widely between and within sponsor types during the study period (October 1, 2013, through January 31, 2016; Figure 1). Insurance companies aired the majority of all advertisements over the 3 open enrollment periods with an average of 169 ad airings per week compared with 61 and 46 airings per week for healthcare.gov and kynect, respectively. Insurance companies aired considerably more ads per week during the 2015 (231 airings) and 2016 (180 airings) open enrollment periods than during 2014 (132 airings). Kynect advertising fell slightly from 2014 (59 airings) to 2015 (52 airings) before a substantial decline for the 2016 open enrollment period (13 airings). In the 2016 open enrollment period, kynect advertising fell from an average of 19 airings per week during the first 9 weeks to none during the final 4 weeks. Furthermore, healthcare.gov advertising fell from a high of 92 airings during the 2014 open enrollment period to only 37 and 23 airings per week during the 2015 and 2016 open enrollment periods, respectively, although these were primarily aired in border markets (Nashville, Cincinnati, Knoxville, and Harrisburg) and likely were not targeting Kentucky specifically. Insurance agencies (20 airings), other state governments (19 airings), and nonprofits (6 airings) each averaged 20 or fewer airings per week over the 3 open enrollment periods.

**Figure 2:**
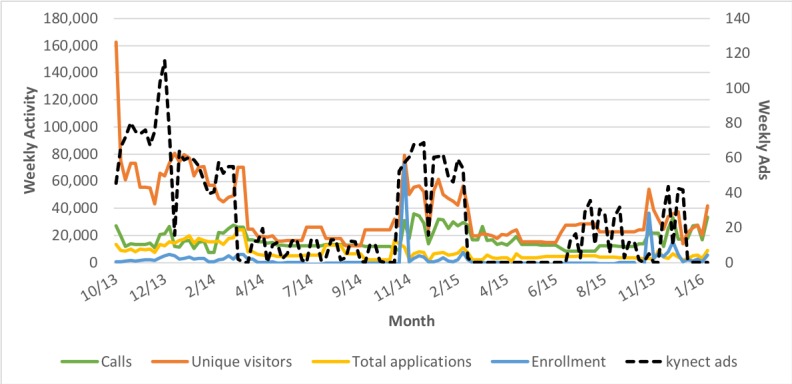
Weekly information-seeking and enrollment outcomes, Kentucky, October 1, 2013-January 31, 2016.

Table 1 presents weekly averages for each information-seeking and enrollment outcome during and outside of open enrollment. Figure 2 shows the weekly trends for calls, unique visitors to the kynect website, total applications, and enrollment. Qualified life events that trigger a special enrollment period (marriage, birth of a child, change in job, change in income, etc) outside of open enrollment provide a baseline level of activity. Calls to the kynect call center were approximately 60% higher during open enrollment, and Web traffic outcomes more than doubled. Similarly, application activity more than doubled through Web and slightly less than doubled in total during open enrollment. Weekly net enrollment in qualified health plans was generally negative outside of open enrollment periods, indicating that disenrollment due to changes in job-related or spousal coverage and nonpayment of premiums outweighed new enrollments outside of open enrollment.

Multimedia Appendix 3 contains linear regression results for our 4 information-seeking behavior outcomes—calls to the kynect call center and page views, visits, and unique visitors for the kynect website. The key measure of dose-response is the marginal effect of advertising volume during open enrollment, which is the sum of the coefficients for the relevant main effect (sponsor type weekly ad volume) and the differential effect (sponsor type weekly ad volume×open enrollment indicator) for each outcome. The marginal effects of kynect advertising during open enrollment are positive and significant for page views, visits, and unique visitors. Specifically, each additional kynect ad airing per week during open enrollment was associated with approximately 8000 additional page views (7972.9, *P*=.001), 400 additional visits (390.2, *P*=.003), and 400 additional unique visitors (387.5, *P*<.001) to the kynect website. There is no evidence of a dose-response effect for advertising by healthcare.gov, insurance companies, and insurance agencies for these outcomes (all marginal effects are not statistically significant for these sponsor types). In contrast, advertising by nonprofits seemed to drive traffic away from kynect resources (calls: −197.6, *P*=.02; page views: −30,061.5, *P*=.03). Advertising by other state governments was associated with increased calls (215.9, *P*<.001) to kynect but no change in Web traffic. Also, we found that the week of Thanksgiving was strongly associated with lower information-seeking activity across all outcomes (calls: −9058.8, *P*=.002; page views: −774,472.7, *P*<.001; visits: −39,814.8, *P*<.001; unique visitors: −23,955.8, *P*<.001) and that the week of Christmas was associated with lower call volume (−8375.9, *P*=.003).

The dose-response effect of kynect advertising on these outcomes was robust to dropping the first week of open enrollment for 2014 (not shown), when the number of unique visitors was at its highest point in the data. The relationship between the kynect advertising volume during open enrollment and calls remained not significant. The relationship (marginal effect) between the kynect advertising volume during open enrollment and page views (7374.7, *P*=.001), visits (347.9, *P*=.005), and unique visitors (357.7, *P*<.001) remained stable and highly significant. We also assessed whether dropping the 2014 open enrollment period altogether (not shown) substantially changed our findings over concerns about the 2014 period being different and driving our overall findings with the Marketplace being new (and, thus, advertising offering novel information). The relationship between kynect advertising volume during open enrollment and calls again was not statistically significant. The relationships (marginal effects) between kynect advertising volume during open enrollment and page views (8705.4, *P*=.02), visits (378.1, *P*<.001), and unique visitors (259.8, *P*<.001) were again similar.

**Table 1:** Weekly averages for information-seeking and enrollment outcomes by open enrollment, Kentucky, October 1, 2013-January 31, 2016.

Outcome		During open enrollment, mean (range)	Outside of open enrollment, mean (range)
**Information-seeking behavior**			
	Calls	21,348 (7724 to 35,905)	13,045 (8470 to 26,634)
	Page views	1,791,512 (471,996 to 3,140,745)	715,252 (473,532 to 1,244,794)
	Visits	94,990 (28,312 to 234,711)	42,477 (27,158 to 62,759)
	Unique visitors	54,525 (13,881 to 162,774)	21,147 (12,621 to 31,888)
**Enrollment activity**			
	Web-based applications	8032 (400 to 23,378)	3290 (148 to 12,707)
	Total applications	10,095 (775 to 24,075)	5179 (949 to 12,792)
	Enrollment	5160 (562 to 73,729)	−430 (−6489 to 2600)

Multimedia Appendix 4 contains linear regression results for our 3 enrollment activity outcomes—number of applications completed through Web, total number of applications completed, and net enrollment in qualified health plans. Again, calculating the marginal effects, we found that each additional kynect ad airing per week during open enrollment was associated with approximately 60 additional applications for coverage completed through Web (61.4, *P*=.03) and more than 55 additional total applications completed (55.9, *P*=.03). Each additional ad per week by insurance agencies during open enrollment was associated with increased Web-based applications (111.4, *P*=.02). Each additional ad per week by other state governments during open enrollment was associated with fewer Web-based applications (−122.8, *P*=.001) and in total (−82.1, *P*=.01). All other marginal effects were not statistically significant.

The week of Thanksgiving was associated with a lower volume of applications completed—both Web based (−3434.1, *P*=.03) and in total (−4523.4, *P*<.001)—mirroring what we observed for information-seeking behavior. The percentage of variation in these outcomes explained by our models was considerably lower—ranging from 56% to 58%—than for the information-seeking outcomes (75% to 81%). This is not surprising given that we would not expect advertising to immediately result in enrollment in the same way that we would for information-seeking behavior. Submitting an application for enrollment requires the consumer to provide tax or income information and selecting a Marketplace plan, if initially deemed not to be Medicaid eligible; this decision may require consideration of various plan options over a longer time period and consultation with a spouse.

We again assessed whether the dose-response effect of kynect advertising on these outcomes was robust to dropping the first week of open enrollment for 2014 or the 2014 open enrollment altogether (not shown). For the former, the relationship (marginal effect) of kynect advertising volume during open enrollment with applications completed through Web (57.3, *P*=.04) and total applications (50.8, *P*=.04) was slightly lower in magnitude but similar. We again found no relationship between kynect advertising volume and net enrollment in qualified health plans.

**Table 2:** Weekly activity attributable to kynect and insurance company advertising, Kentucky, 2014 and 2015 open enrollment periods.

Outcome		Actual, n	Model-based predictions				
			Prediction (with observed advertising), n^a^	Counterfactual (no kynect advertising), n^a^	Change attributable to kynect advertising, n (%)^b^	Counterfactual (no insurance company advertising), n^a^	Change attributable to insurance company advertising, n (%)^c^
**Information-seeking behavior**							
	Page views	1,978,749	1,925,863	1,474,730	451,133 (23)	1,804,879	120,984 (6)
	Visits	105,618	101,616	79,538	22,078 (22)	98,061	3555 (3)
	Unique visitors	61,838	58,964	37,037	21,927 (37)	55,242	3722 (6)
**Enrollment activity**							
	Web-based applications	9643	9050	5576	3474 (38)	11,759	–2709 (–30)
	Total applications	11,832	11,232	8072	3160 (28)	12,516	−1283 (−11)

^a^Represent model-based predictions (using the model results shown in Multimedia Appendix 3 and Multimedia Appendix 4) of the weekly outcomes. These comparisons use the 2014 and 2015 open enrollment periods to estimate how much activity was attributable to advertising. Calls and Marketplace enrollment are not included because neither kynect nor insurance company advertising during open enrollment had a significant effect on these outcomes.

^b^Difference (%) between "Prediction (with observed advertising)" and "Counterfactual (no kynect advertising)".

^c^Difference (%) between "Prediction (with observed advertising)" and "Counterfactual (no insurance company advertising)".

For the latter, the relationship (marginal effect) between the kynect advertising volume during open enrollment and all 3 enrollment outcomes was not statistically significant. This is not surprising given the weaker relationship that we observed in our main results for the enrollment outcomes compared with the information-seeking outcomes, particularly in light of the potential for automatic re-enrollment driving activity when the 2014 open enrollment period is excluded from the analysis.

Table 2 contains estimates of information-seeking and enrollment activity attributable to kynect and insurance company advertising, using our models to predict the average weekly activity given observed advertising and 2 counterfactuals (no kynect advertising, no insurance company advertising). With the substantial drop and eventual elimination of kynect advertising during the 2016 open enrollment period, we used the 2014 and 2015 open enrollment periods as the setting for this portion of the analysis, given the relative stability in state-sponsored advertising and political support for kynect (base scenario). Based on our estimates, kynect advertising was associated with approximately 23% of page views, 22% of visits, and 37% of unique visitors to the kynect website compared with only single digits (6%, 3%, and 6%, respectively) for insurance company advertising. Approximately 38% of Web-based and 28% of total applications were associated with kynect advertising. Insurance company advertising was estimated to be a negative contributor to applications (−30% of Web-based and −11% of total applications), likely because it drove consumers to carriers to enroll in off-exchange plan offerings rather than directing them to the Marketplace [21].

## Discussion

Our study is the first to use weekly variation in television advertising to explain consumer engagement with the Marketplace. Specifically, we found significant dose-response effects of the weekly state-sponsored advertising volume during open enrollment on information-seeking behavior (page views, visits, and unique visitors) and enrollment activity (applications). This research offers new evidence of the importance of health insurance advertising in the context of the ACA. Our findings differ from other research on health insurance marketing, which generally find small effects. For example, a recent analysis of the Medicare Advantage market, where television advertising for a large national insurer was found to have a limited effect on encouraging switching from traditional Medicare to Medicare Advantage [22]. This is in contrast to analyses of the first open enrollment period for the ACA in which significant positive effects of television advertising were found [10,11]. Covered California, the state-based Marketplace for the state bearing its name, claims a “more than three-to-one return on investment” on its marketing spending, which “likely lower[ed] premiums by 6 to 8 percent” through improved enrollment and lower risk scores for its enrolled population [12,23].

However, the study is not without limitations. The lack of outcome data at the county or media market level necessitated the construction of statewide population-weighted averages of advertising volume, which reduced variation in our explanatory variables of interest. This is a particular issue owing to border markets where federal and other state advertising spillover into the state are averaged into the statewide advertising measures. Weekly counts of advertising are a cruder measure of exposure than the more desirable gross rating points (which we do not have), which incorporates the ratings for the program during which an ad was aired to provide a more detailed description of the potential reach [18,19]. It is also important to note that Medicaid enrollment is not reflected in our enrollment outcome. Kentucky saw a 6.4 percentage point increase in Medicaid coverage from 2012 to 2015 compared with only a 0.9 percentage point increase in individual nongroup coverage over the same period [24]. The state had approximately half a million enrollees in its Medicaid expansion versus just over 100,000 Marketplace enrollees at its peak in 2015 [24,25]. Although we found no relationship between state-sponsored advertising and enrollment in Marketplace plans, we likely would have observed one if our enrollment measure was more broad (Marketplace plus Medicaid) as it was for the application outcomes that do capture those consumers who may ultimately enroll in Medicaid. Indeed, in a national county-level analysis, a strong relationship was observed between the volume of state-sponsored advertising and Medicaid enrollment [10]. There is no obvious control group because all state-based Marketplaces carry distinct branding and operate in varying political and health insurance market contexts, so we used an interrupted time series approach as the next best option to counteract this weakness to the extent possible, using the time periods outside of open enrollment to establish a baseline level of activity to compare with the open enrollment periods. While dose-response evidence helps us come closer to understanding this relationship, it is not definitive causal evidence. We are measuring two contemporaneous phenomena as including lagged and year-specific effects stretched beyond the limitations of what our data would allow. However, despite these limitations, we were still able to detect strong relationships between the volume of television advertising and our outcomes, reinforcing the role advertising plays in increasing consumer engagement and maximizing potential enrollment. Kentucky ran a highly successful and well-designed campaign in the context of a state-based Marketplace. Therefore, the strength of the dose-response relationship may not generalize to other state-based Marketplaces or those that are federally run due to variation in messaging strategy, demographics of the Marketplace eligible population, Medicaid expansion status, and other factors. This study does not specifically address the message content and tagging (ie, phone numbers, URLs) of health insurance advertising by various ad sponsors, which may be related to differences in their effectiveness in driving Marketplace activity.

Finally, we only assessed television advertising, which took place in the context of larger media campaigns by some (but not all) sponsors. Television is still the dominant news source and advertising medium, despite the growth of digital advertising in recent years [26,27]. It is not feasible from a data availability standpoint to control for all potential advertising channels (eg, radio, digital, print, and out-of-home); however, to the extent that the patterns and targeting of advertising in other channels did not substantially diverge over time from that of the television advertising, this is not an issue for our analysis.

The decision to eliminate television advertising and cut funding for advertising by 90% overall in the federally run Marketplace for the 2018 open enrollment period was justified by administration representatives citing the “diminishing returns” to advertising demonstrated during previous years [1]. However, this has been disputed by members of the Obama administration [28]. The reported shift to a heavier focus on digital advertising may allow for reaching a sizable population at a fraction of the cost but should not be expected to provide the same impact as a television campaign, as TV still garners a majority of viewing time [29]. This conclusion is supported not only by this study but also by evidence from an ongoing national health education campaign [30]. In addition, our results indicate that we should not expect insurance company advertising to help fill the void in terms of generating consumer engagement with the Marketplace without modification of their ad content (eg, explicitly directing consumers to healthcare.gov rather than their own websites). Finally, our findings also speak to another decision related to the fifth open enrollment period under the Trump administration. The large drops in activity that we observed during the week of Thanksgiving suggest that shortening the open enrollment period from 90 to 45 days (from November 1 to December 15) could have further negative consequences, as the Thanksgiving week represents a substantial proportion of the shortened enrollment period.

Approaching the recently completed fifth open enrollment period, 4 in 10 uninsured Americans were still unaware of the Marketplace and a majority of prior Marketplace enrollees were uncertain about the timing of open enrollment [31,32]. Our findings from Kentucky indicate that state-sponsored advertising appears to be highly correlated with traffic to the Marketplace website and applications for coverage—with the kynect television campaign associated with nearly 40% of unique visitors and Web-based applications. This resonates with prior research, albeit limited, that finds a strong link between exposure to Marketplace advertising with perceptions and knowledge about the ACA, improved (lower) risk pools, and reductions in the uninsured rate [8-12]. Insurance company advertising seemed to play a limited role in driving consumers to engage with the Marketplace, likely because their advertising was more geared toward creating brand preference and gaining market share among enrollees (in the Marketplace, but also in employer-sponsored plans) rather than serving as a call to action. These findings raise questions about what role government—as a social institution, rather than a legislative and administrative body—should play in promoting its programs to Americans, particularly for an issue as important and politically charged as health care.

## Appendix

Multimedia Appendix 1 Ad sponsors and sponsor type, Kentucky, October 1, 2013–January 31, 2016.

Multimedia Appendix 2 Correlation matrix.

Multimedia Appendix 3 Information-seeking behavior models, Kentucky, October 1, 2013–January 31, 2016.

Multimedia Appendix 4 Enrollment activity models, Kentucky, October 1, 2013–January 31, 2016.

## Abbreviations

ACA: Affordable Care Act
CMAG: Campaign Media Analysis Group
KHBE: Kentucky Health Benefit Exchange
