# Supplementary material for: Television Advertising and Health Insurance Marketplace Consumer Engagement in Kentucky: A Natural Experiment
Source: J Med Internet Res. 2018 Oct 25;20(10):e10872. doi: 10.2196/10872 (PMC6234351; doi:10.2196/10872)
Supplement: Multimedia Appendix 1 [file jmir_v20i10e10872_app1.pdf]

Appendix 1. Ad sponsors and sponsor type, Kentucky, October 1, 2013–January 31, 2016

Sponsor Type	Ad Sponsor(s)
kynect	kynect
healthcare.gov	U.S. Department of Health and Human Services
Insurance companies	Aetna, Blue Cross/Blue Shield – Anthem, Blue Cross/Blue Shield of Tennessee, Care Source, Cigna, Coventry Health Care, Crestpoint Health, Farm Bureau Insurance, Gateway Health Plan, Healthspan, Humana, In Health Ohio, Kentucky Health Cooperative, MDwise, Medical Mutual, Medigold, Molina Healthcare, OptumHealth, Passport Health Plan, RiverLink Health, Transamerica, TRH Health Plans, UniCare, United Healthcare, WellCare Health Plans, Windsor Health Plan, Inc.
Insurance agencies	Affordable Care Health Insurance, American Exchange, Enroll Health, Health Insurance Hotline, Healthmarkets Insurance Agency, HealthTN, iCan, Insurance Options, Medicare Health Reform Helpline, Nations Helpline, Prescription Assistance Helpline, Quick Insurance 123, Uninsured Helpline
Nonprofits	Get Covered America, Health Kentucky, Health Plan, Health Plan of West Virginia, National Alliance for Hispanic Health, West Virginia Primary Care Association
Other state governments	Are You Covered Ohio, Cover Virginia, Get Covered Illinois, Ohio for Health, ohioforhealth.org
Excluded[a]	AFLAC (disability insurance), Community Care (provider in West Virginia), Free Rx Network (prescription discount card), Premier Health (provider network in Ohio), Select Advisor (final expense insurance), Simply Healthcare (membership-based provider in Tennessee), TriHealth (provider network in Ohio), VSP Vision Care (vision insurance)

[a] Ad sponsors excluded from our analysis result in the loss of 8,406 of 130,257 (6.5%) individual ad airings represented in the Kantar data set during the study period.
