# Supplementary material for: Television Advertising and Health Insurance Marketplace Consumer Engagement in Kentucky: A Natural Experiment
Source: J Med Internet Res. 2018 Oct 25;20(10):e10872. doi: 10.2196/10872 (PMC6234351; doi:10.2196/10872)
Supplement: Multimedia Appendix 2 [file jmir_v20i10e10872_app2.pdf]

Appendix 2. Correlation Matrix

	Number of kynect ads	Number of insurance company ads	Number of healthcare.gov ads	Number of insurance agency ads	Number of nonprofit ads	Number of other state government ads	Open enrollment period	Week of Thanksgiving	Week of Christmas	Last two weeks before open enrollment	First two weeks of open enrollment	Last two weeks of open enrollment	Number of days in the reporting period
Number of kynect ads	1.0000												
Number of insurance company ads	0.5243	1.0000											
Number of healthcare.gov ads	0.4761	0.1423	1.0000										
Number of insurance agency ads	0.2046	0.5247	0.2505	1.0000									
Number of nonprofit ads	-0.2085	-0.1989	-0.0077	-0.1093	1.0000								
Number of other state government ads	0.2684	0.1957	0.3205	0.3580	-0.0805	1.0000							
Open enrollment period	0.6783	0.5660	0.5910	0.5232	-0.1931	0.3995	1.0000						
Week of Thanksgiving	-0.0014	-0.0750	0.0437	0.0491	-0.0445	0.0643	0.1782	1.0000					
Week of Christmas	0.1695	0.0737	0.1830	0.0589	-0.0137	0.0792	0.1782	-0.0252	1.0000				
Last two weeks before open enrollment	-0.0726	-0.0930	0.2371	0.0305	0.0906	-0.0978	-0.1641	-0.0292	-0.0292	1.0000			
First two weeks of open enrollment	0.1162	-0.0363	0.3671	0.0558	0.0149	0.0777	0.2552	-0.0361	0.2086	-0.0419	1.0000		
Last two weeks of open enrollment	-0.1193	0.2132	-0.0764	0.1968	-0.0191	0.2300	0.2552	-0.0361	-0.0361	-0.0419	-0.0517	1.0000	
Number of days in the reporting period	-0.0742	0.0208	-0.0315	0.1290	-0.0585	0.2666	0.0305	0.0054	0.0054	-0.1860	-0.1505	0.1661	1.0000
