# Supplementary material for: Television Advertising and Health Insurance Marketplace Consumer Engagement in Kentucky: A Natural Experiment
Source: J Med Internet Res. 2018 Oct 25;20(10):e10872. doi: 10.2196/10872 (PMC6234351; doi:10.2196/10872)
Supplement: Multimedia Appendix 3 [file jmir_v20i10e10872_app3.pdf]

Multimedia Appendix 3. Information-seeking behavior models, Kentucky, October 1, 2013–January 31, 2016

	Beta (95% confidence interval)			
**Covariate**	**Calls**	**Page views**	**Visits**	**Unique visitors**
Number of kynect ads	–113.2**	–4,222.7	–156.7	–62.0
	(–170.5, –56.0)	(–9,229.5, 784.0)	(–564.6, 251.2)	(–312.9, 188.9)
Number of kynect ads *x* open enrollment period	86.5	12,195.6**	546.9*	449.6**
	(–1.7, 174.8)	(5,285.4, 19,105.8)	(56.3, 1,037.5)	(146.7, 752.5)
Number of insurance company ads	–8.7**	971.8**	39.7**	19.1**
	(–14.9, –2.4)	(489.6, 1,454.0)	(15.1, 64.4)	(5.6, 32.7)
Number of insurance company ads *x* open enrollment period	21.3*	–258.0	–19.3	1.9
	(4.0, 38.5)	(–2,165.6, 1,649.6)	(–122.8, 84.3)	(–67.7, 71.4)
Number of healthcare.gov ads	101.2	4,661.5	77.0	–123.7
	(–22.5, 224.9)	(–7,084.5, 16,407.4)	(–472.6, 626.5)	(–361.1, 113.8)
Number of healthcare.gov ads *x* open enrollment period	–118.9	–4,796.0	–49.4	145.1
	(–245.5, 7.7)	(–16.727.1, 7,135.0)	(–612.9, 514.2)	(–103.4, 393.6)
Number of insurance agency ads	47.6	–10,825.6**	–137.1	–0.1
	(–57.8, 153.0)	(–17.856.6, –3,794.7)	(–725.4, 451.2)	(368.4, 368.3)
Number of insurance agency ads *x* open enrollment period	–86.8	15,471.9**	241.9	17.3
	(–216.4, 42.8)	(5,104.7, 25,839.1)	(–471.5, 955.3)	(–421.8, 456.3)
Number of nonprofit ads	28.9	–472.5	–81.7	–35.2
	(–40.3, 98.0)	(–2,161.2, 1,216.2)	(–212.2, 48.8)	(–113.7, 43.4)
Number of nonprofit ads *x* open enrollment period	–226.4*	–29,589.0*	–890.2	1.4
	(–411.1, 41.8)	(–56,776.7, –2,401.3)	(–2,379.3, 598.8)	(–1,014.7, 1,017.5)
Number of other state government ads	24.6	1,870.6	210.1*	150.9*
	(–62.0, 111.3)	(–2,379.7, 6,120.8)	(14.6, 405.6)	(37.2, 264.6)
Number of other state government ads *x* open enrollment period	191.2**	–2,651.8	–209.6	–357.1**
	(85.2, 297.3)	(–9,975.0, 4,671.5)	(–593.3, 174.1)	(–612.6, 101.7)
Open enrollment period	6,353.7*	614,110.9	29,021.2	10,977.3
	(167.9, 12,539.5)	(–84,740.1, 1,312,962.0)	(–6,820.7, 64,863.1)	(–9,200.2, 31,154.9)

Week of Thanksgiving	–9,058.8**	–774,472.7**	–39,814.8**	–23,955.8**
	(–14,607.5, –3,510.0)	(–950,214.9, –598,730.5)	(–52,318.2,–27,311.3)	(–31,921.1,–15,990.5)
Week of Christmas	–8,375.9**	–251,521.5	–13,993.2	–381.9
	(–13,893.2, –2,858.7)	(–1,280,231.0, 777,188.4)	(–63,518.5, 35,532.2)	(–37,288.1, 36.524.2)
Last two weeks before open enrollment	–116.1	6,629.0	–4,710.7	–2,518.2
	(–2,327.3, 2,095.2)	(–194,436.8, 207,694.7)	(–23,833.6, 14,412.2)	(–14,564.8, 9,528.4)
First two weeks of open enrollment	1,512.3	404,244.2	34,109.6*	27,707.9**
	(–1,942.6, 4,967.1)	(–44,398.4, 852,886.8)	(6,398.1, 61,821.1)	(8,129.9, 47,285.9)
Last two weeks of open enrollment	4,409.7	939,001.5**	40,720.5*	21,530.9*
	(–1,091.7, 9,911.1)	(301,538.1, 1,576,465.0)	(4,544.8, 76,896.3)	(1,342.0, 41,719.8)
Number of days in the reporting period	–3,104.3*	–240,963.4	–38.921.5*	–24,430.7
	(–6,060.5, –148.1)	(–790,110.5, 308,183.7)	(–76,014.4, –1,828.7)	(–50,480.0, 1,618.6)
Number of observations (state-weeks)	122	122	122	122
R^2	0.75	0.81	0.79	0.80

* P<.05, ** P<.01
