# Supplementary material for: Television Advertising and Health Insurance Marketplace Consumer Engagement in Kentucky: A Natural Experiment
Source: J Med Internet Res. 2018 Oct 25;20(10):e10872. doi: 10.2196/10872 (PMC6234351; doi:10.2196/10872)
Supplement: Multimedia Appendix 4 [file jmir_v20i10e10872_app4.pdf]

Multimedia Appendix 4. Enrollment activity models, Kentucky, October 1, 2013–January 31, 2016

	Beta (95% confidence interval)		
**Covariate**	**Online applications**	**Total applications**	**Enrollment**
Number of kynect ads	19.0	29.0	108.9
	(–51.0, 88.9)	(–25.9, 83.9)	(–17.5, 235.3)
Number of kynect ads *x* open enrollment period	42.4	26.8	–128.9
	(–49.1, 134.0)	(–48.6, 102.3)	(–272.9, 15.2)
Number of insurance company ads	1.5	–4.0	6.0
	(–11.0, 14.1)	(–12.1, 4.0)	(–0.9, 12.9)
Number of insurance company ads *x* open enrollment period	–18.2	–4.1	12.3
	(–45.4, 8.9)	(–25.0, 16.8)	(–13.1, 37.7)
Number of healthcare.gov ads	51.5	–26.1	72.1
	(–95.5, 198.4)	(–123.5, 71.3)	(–18.6, 162.8)
Number of healthcare.gov ads *x* open enrollment period	–39.5	34.2	–72.4
	(–189.2, 110.3)	(–66.2, 134.5)	(–163.2, 18.5)
Number of insurance agency ads	–107.3	–1.7	–150.8
	(–327.1, 112.5)	(–166.7, 163.3)	(–327.9, 26.3)
Number of insurance agency ads *x* open enrollment period	218.7	78.0	105.6
	(–19.3, 456.6)	(–105.5, 261.5)	(–78.8, 290.0)
Number of nonprofit ads	–61.7**	–27.7	26.1
	(–90.5, –32.9)	(–60.5, 5.1)	(–16.7, 68.9)
Number of nonprofit ads *x* open enrollment period	–63.9	–208.5	–98.0
	(–340.9, 213.1)	(–450.0, 33.0)	(–394.4, 198.5)
Number of other state government ads	–116.3**	–59.7*	–46.6
	(–179.3, –53.2)	(–115.0, –4.4)	(–104.2, 11.0)
Number of other state government ads *x* open enrollment period	–6.5	–22.4	18.0
	(–99.3, 86.3)	(–103.2, 58.3)	(–98.7, 134.7)
Open enrollment period	3,307.5	3,851.8	4,447.0
	(–5,673.5, 12,288.5)	(–3,368.4, 11,072.1)	(–2,833.5, 11,727.3)

Week of Thanksgiving	–3,434.1*	–4,523.4**	–7,897.1
	(–6,464.3, –404.0)	(–6,958.5, –2,088.3)	(–21,204.2, 5,410.0)
Week of Christmas	–1,057.4	–2,669.1	–1,390.8
	(–8,622.7, 6,507.8)	(–10,054.2, 4,715.9)	(–4,857.0, 2,075.4)
Last two weeks before open enrollment	–604.6	–990.2	–122.9
	(–3,486.6, 2,277.3)	(–3,125.7, 1,145.2)	(–5,603.2, 5,357.4)
First two weeks of open enrollment	814.1	537.5	17,592.3
	(–2,969.1, 4,597.2)	(–4,240.1, 5,315.1)	(–3,502.8, 38,687.4)
Last two weeks of open enrollment	4,266.3	5,364.9	214.0
	(–3,009.5, 11,542.1)	(–643.3, 11,373.0)	(–5,254.9, 5,682.9)
Number of days in the reporting period	–835.6	–35.8	15,295.2
	(–4,682.4, 3,011.1)	(–5,057.5, 4,985.9)	(–1,127.1, 31.717.5)
Number of observations (state-weeks)	122	122	122
R^2	0.56	0.58	0.56

* P<.05, ** P<.01
